# Cystic Renal Cell Carcinoma Subtype in Pediatric Patient: A Case Report

**DOI:** 10.7759/cureus.28331

**Published:** 2022-08-24

**Authors:** Radhi A Alshehri, Suliaman Alaqeel, Shahad H AlOtaiby, Ahmad Aldraihem

**Affiliations:** 1 Department of Medical Imaging, King Fahad Medical City, Riyadh, SAU; 2 Department of Pediatric Surgery, Ministry of National Guard Health Affairs, Riyadh, SAU; 3 Scientific Writing, King Fahad Medical City, Riyadh, SAU

**Keywords:** tumor, wilms, biopsy, pediatrics, carcinoma, rcc, cell, renal, cystic

## Abstract

A 10-year-old boy presented with a right flank mass. Computed tomography, ultrasound scan, and magnetic resonance imaging confirmed the presence of a multiloculated cystic mass. After right kidney nephrectomy, the biopsy proved the diagnosis of cystic renal cell carcinoma, which is a rare subtype of renal cell carcinoma in the pediatrics age group. The knowledge about this incidence can optimize the investigations, management, and outcomes.

## Introduction

Renal cell carcinoma (RCC) is mostly detected in the advanced age group, usually between 50 to 70 years of age, and approximately 90-95% of cases of this disease are in this age group. RCC is infrequently encountered in children, with an annual incidence of 4/1,000,000 children [1]. RCC tumors are rare in childhood and are estimated to be from 1.8% to 6.3% of all malignant renal tumors [2,3]. The histological classification of RCCs is extremely important, considering the significant implications of the subtypes in the prognosis and treatment of these tumors [4]. However, the cystic subtype is an extremely infrequent lesion in the adult with a male/female ratio of 3:1 at the mean age of 50 years [5]. RCC is usually misdiagnosed as a benign renal cyst, and it also shares similar clinical presentation and imaging features to Wilms tumor [6]. RCC carries an excellent prognosis and can be completely cured by complete surgical resection, which is the primary treatment [7]. Here, we present a case of a 10-year-old patient with RCC detected incidentally in King Fahad Medical City, Saudi Arabia.

## Case presentation

A 10-year-old boy with an unremarkable past medical history and normal growth percentile on regular visits with primary care physician presented with a right flank mass noted by the mother. The patient's abnormal physical exam findings were a right flank palpable non-tender firm mass. The rest of the complete physical exam was normal. Initial laboratory workup included a complete blood count, urinalysis, and renal function tests, which were within normal limits except for hematuria. No family history of a similar presentation was reported.

**Figure 1:**
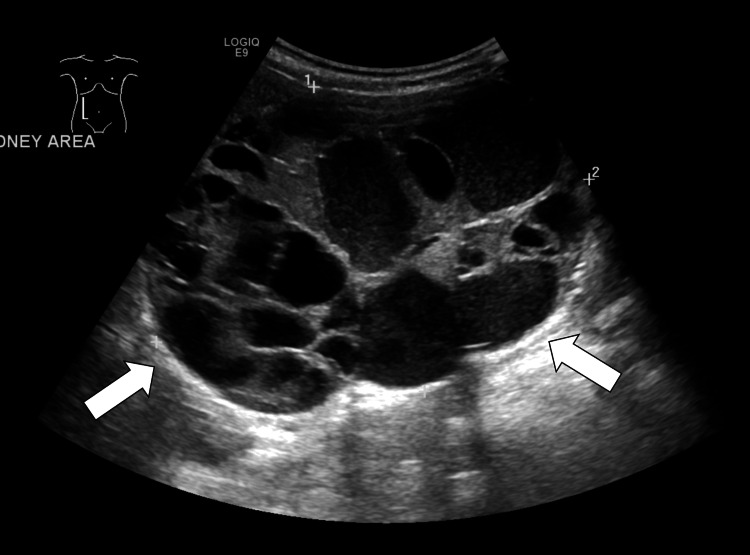
Ultrasound scan over the right kidney shows multiple cystic lesions with multiple loculations with no evidence of discernible solid component (arrows)

**Figure 2:**
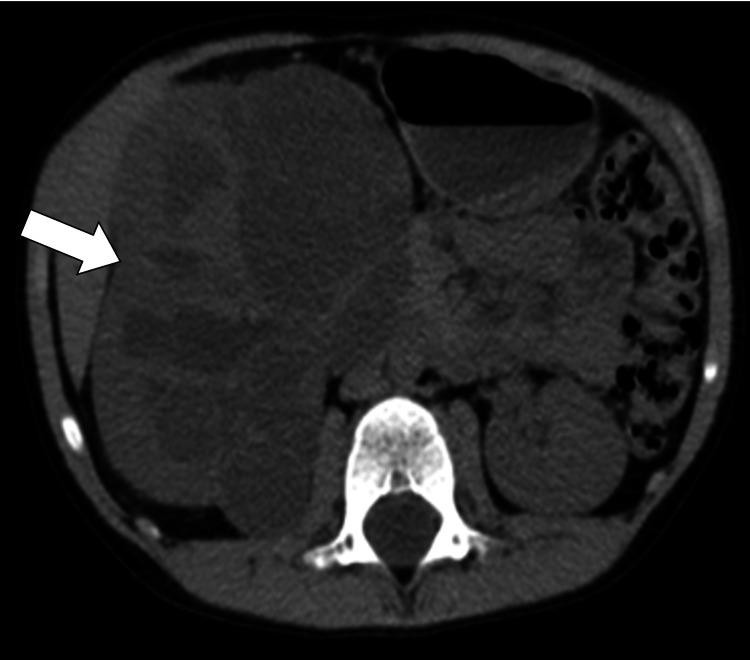
Axial non-enhanced CT scan showing multiloculated well defined soft tissue lesion arising from the right kidney measuring 11x8x7.5 cm (arrow)

**Figure 3:**
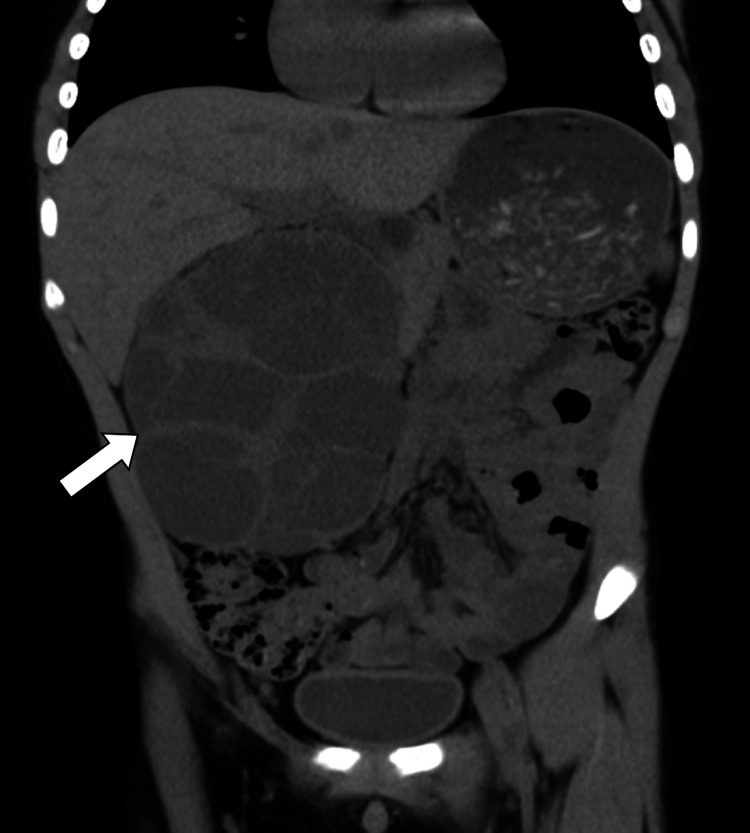
Coronal non-enhanced CT image showing clearly the multicystic nature of the large right kidney mass (arrow)

**Figure 4:**
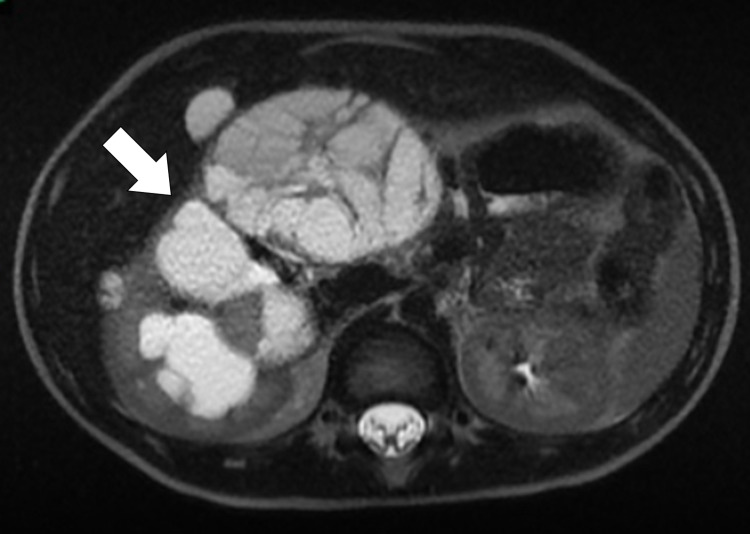
Axial T2 weighted MRI image demonstrates a well-circumscribed, encapsulated mass consisting of multiple cysts with variably enhancing septa soft tissue without vascular or adjacent organ invasion

An initial screening ultrasound (Figure 1) was done, showing the right cystic mass with solid components raising the susception of a complex renal cyst. Cross-sectional CT scan imaging (Figures 2-3) and MRI scan (Figure 4) were done, also showing a predominantly cystic mass with some solid components raising the concern for malignant degeneration.

**Figure 5:**
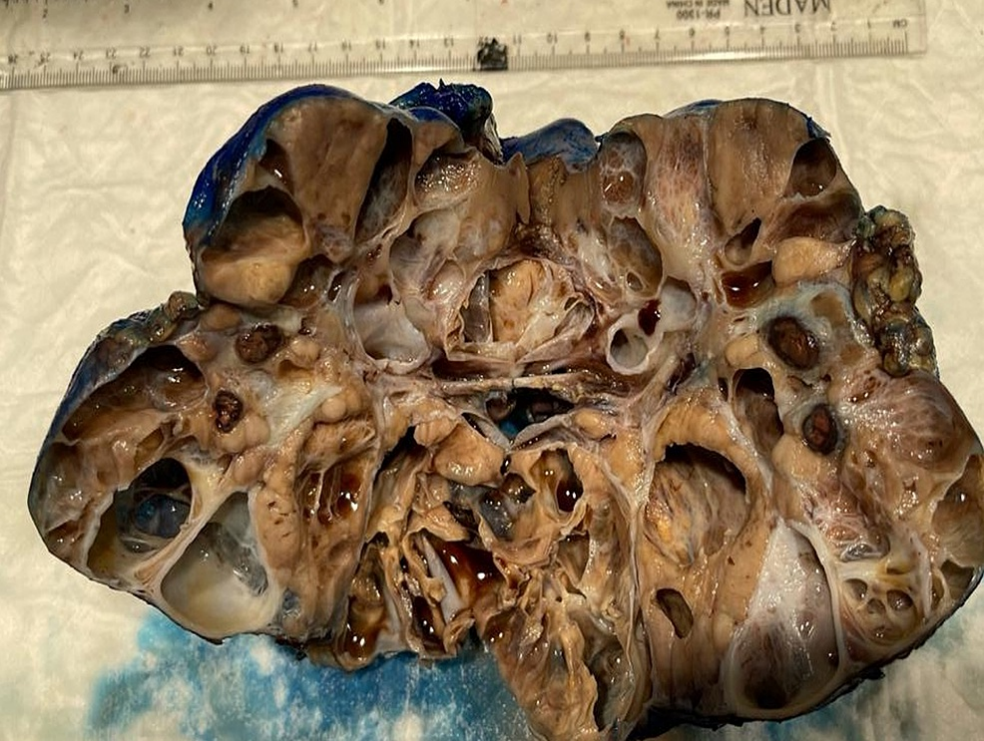
Gross image post radical right kidney nephrectomy showing large multilocular cystic renal cell carcinoma

Initially, an image-guided biopsy was performed; however, due to sample inadequacy, no diagnosis could be reached. The medical team decided to take the patient for an open incisional biopsy to include both the cystic and solid components in the biopsy. A frozen section by open surgical biopsy was not conclusive, and the pathologist required a permanent section to determine prior to performing a nephrectomy. After confirming the neoplastic nature of the renal cystic mass, the patient was taken for right nephrectomy through a right supra-umbilical transverse incision. Complete gross excision was achieved with no intraoperative or postoperative complication (Figure 5). As a result, a tumor board discussion was made and indicated no need for chemotherapy, radiotherapy, or any adjuvant therapies during the treatment course period. 

Outcomes and follow-up

No evidence of local recurrence was noted after 18 months as there was no distant or local metastasis. Also, after three months of follow-up in the clinic, the patient was symptom-free without any complaint, the laboratory results of the renal function tests were within normal limits, and the preoperative technetium-99m mercaptuacetyltriglycine scan (MAG3) demonstrates normal functioning kidneys despite the presence of the tumor. The patient was scheduled for interval follow-up and imaging after three and six months, and there were no complications. Routine long-term follow-ups were scheduled with the patient for health monitoring.

No further family screening was requested by the medical team.

## Discussion

King Fahad Medical City is a tertiary and referral hospital where a lot of cases get referred to, and here we reported a rare case of RCC in pediatric patients. RCC accounts for only 3% of malignant renal tumors, and it's far more common in adults; however, renal cell cancer (RCC) does occur in older children and adolescents [8]. 

Cystic renal cell carcinoma subtype is a very rare type of renal malignancy in the pediatric age group. It could be locally more advanced than the adult age group at presentation [8]. Generally, there is no sex predominance for this renal tumor type in children found in the literature. The most common form of presentation of RCC in children is macroscopic hematuria and abdominal or flank pain. Other less frequent symptoms are palpable abdominal mass, anemia, and fever [8]. Since cystic RCC and the other differentials share similar characteristics on imaging, preoperative differentiation​ may not be possible. It has mainly four differential diagnoses that have a strikingly similar appearance in the gross examination, including cystic nephroma, cystic partially differentiated nephroblastoma, multilocular cystic renal cell carcinoma, and cystic hamartoma of the renal pelvis. The definite diagnosis maybe not be established unless the pathological workup is conducted either by biopsy or complete surgical excision [9].

The preoperative classification has low practical importance in management as all cystic renal tumors undergo surgical excision given their intrinsic resistance to chemotherapy and radiation therapy [10]. However, all management decisions are made by a multidisciplinary tumor board, including pediatric surgeons, pediatric oncologists, and radiation oncologists. The accurate RCC classification may have important implications for patients and their families for the determination of prognostic risk stratification, targeted therapeutics selection, and identification for genetic testing [11]. The World Health Organization (WHO) classification of kidney tumors recognizes multilocular cystic renal cell carcinoma (MCRCC) as a rare variant of clear cell renal cell carcinoma with a good prognosis [11,12]. The subtype classification of this tumor is extremely rare in this age group and should be considered within the differential diagnosis of the common multi-cystic renal tumors in the pediatric age group.

## Conclusions

We reported a rare incidence of cystic subtype renal cell carcinoma in a 10-year-old boy, which is an extremely rare subtype in this age group. Cystic Wilms tumor, cystic nephroma, and cystic partially differentiated nephroblastoma should be considered as differential diagnoses with these radiological features. Consideration of RCC in children older than five years is very important since the diagnostic and therapeutic approach differs from that for Wilms tumor. The technical difficulty of cystic tumor sampling may reduce the value of image-guided and open surgical biopsy due to the difficulty in obtaining an appropriate solid component to establish the final diagnosis, which may not be made until the complete surgical resection is performed. The subtypes of multicystic renal tumors can be differentiated only by histopathology. This case contributes to the incidence of such case reports published in the literature and adds weight to this rare case of cystic RCC in the pediatric population.

## References

[1] Chung EM, Graeber AR, Conran RM (2016). Renal tumors of childhood: radiologic-pathologic correlation part 1. The 1st decade: from the radiologic pathology archives. Radiographics.

[2] Indolfi P, Terenziani M, Casale F (2003). Renal cell carcinoma in children: a clinicopathologic study. J Clin Oncol.

[3] Abdellah A, Selma K, Elamin M (2015). Renal cell carcinoma in children: case report and literature review. Pan Afr Med J.

[4] Lopez-Beltran A, Scarpelli M, Montironi R, Kirkali Z (2006). 2004 WHO classification of the renal tumors of the adults. Eur Urol.

[5] Prasad SR, Humphrey PA, Catena JR (2006). Common and uncommon histologic subtypes of renal cell carcinoma: imaging spectrum with pathologic correlation. Radiographics.

[6] Muglia VF, Prando A (2015). Renal cell carcinoma: histological classification and correlation with imaging findings. Radiol Bras.

[7] Chen S, Jin B, Xu L (2014). Cystic renal cell carcinoma: a report of 67 cases including 4 cases with concurrent renal cell carcinoma. BMC Urol.

[8] Estrada CR, Suthar AM, Eaton SH, Cilento BG Jr (2005). Renal cell carcinoma: Children's Hospital Boston experience. Urology.

[9] Kurian JJ, Jehangir S, Korula A (2018). Multiloculated cystic renal tumors of childhood: has the final word been spoken. J Indian Assoc Pediatr Surg.

[10] Silberstein J, Grabowski J, Saltzstein SL, Kane CJ (2009). Renal cell carcinoma in the pediatric population: results from the California Cancer Registry. Pediatr Blood Cancer.

[11] Udager AM, Mehra R (2016). Morphologic, molecular, and taxonomic evolution of renal cell carcinoma: a conceptual perspective with emphasis on updates to the 2016 World Health Organization classification. Arch Pathol Lab Med.

[12] Eble JN, Bonsib SM (1998). Extensively cystic renal neoplasms: cystic nephroma, cystic partially differentiated nephroblastoma, multilocular cystic renal cell carcinoma, and cystic hamartoma of renal pelvis. Semin Diagn Pathol.

